# Clinical application of intraoperative ultrasound superb microvascular imaging in brain tumors resections: contributing to the achievement of total tumoral resection

**DOI:** 10.1186/s12880-024-01321-5

**Published:** 2024-06-11

**Authors:** Siman Cai, Hao Xing, Yuekun Wang, Yu Wang, Wenbin Ma, Yuxin Jiang, Jianchu Li, Hongyan Wang

**Affiliations:** 1 Department of Medical Ultrasound, Peking Union Medical College Hospital, Chinese Academy of Medical Science and Peking Union Medical College, Beijing, 100730 China; 2 Department of the Neurosurgery Department, Peking Union Medical College Hospital, Chinese Academy of Medical Science and Peking Union Medical College, Beijing, 100730 China

**Keywords:** Glioma, Superb microvascular imaging (SMI), Intraoperative imaging, Ultrasound, Gross-total resection (GTR).

## Abstract

**Background:**

To investigate whether the intraoperative superb microvascular imaging(SMI) technique helps evaluate lesion boundaries compared with conventional grayscale ultrasound in brain tumor surgery and to explore factors that may be associated with complete radiographic resection.

**Methods:**

This study enrolled 57 consecutive brain tumor patients undergoing surgery. During the operation, B-mode and SMI ultrasound evaluated the boundaries of brain tumors. MRI before and within 48h after surgery was used as the gold standard to evaluate gross-total resection(GTR). The ultrasound findings and GTR results were analyzed to determine the imaging factors related to GTR.

**Results:**

A total of 57 patients were enrolled in the study, including 32 males and 25 females, with an average age of 53.4 ± 14.1 years old(range 19 ~ 80). According to the assessment criteria of MRI, before and within 48 h after the operation, 37(63.9%) cases were classified as GTR, and 20(35.1%) cases were classified as GTR. In comparing tumor interface definition between B-mode and SMI mode, SMI improved HGG boundary recognition in 5 cases(*P* = 0.033). The results showed that the tumor size ≥ 5 cm and unclear ultrasonic boundary were independent risk factors for nGTR (OR>1, *P*<0.05).

**Conclusions:**

As an innovative intraoperative doppler technique in neurosurgery, SMI can effectively demarcate the tumor’s boundary and help achieve GTR as much as possible.

## Introduction

For brain tumors, gross-total resection (GTR) of lesions has always been challenging and crucial, and accurate localization of lesions has become the key to the success of surgery. Compared with non-gross-total resection (nGTR), GTR can not only prolong overall survival but also improve seizure control and reduce the incidence of malignant transformation [1]. During neurosurgery operations, the location of the lesion and the extent of resection should be considered. GTR depends on the precise localization of the brain tumor and accurate identification of tumor boundaries and residual tumors. However, infiltrative growth is a typical growth pattern of most malignant tumors, mostly without clear demarcation from normal brain tissue, and is often accompanied by peripheral edema, resulting in surgical difficulty in completely removing lesions [2]. Its infiltrative nature poses a challenge to correctly detecting the tumor border, and maximal resection of the tumor without invading the surrounding functional area remains a complex problem to solve.

Currently, neuronavigation systems are widely used in the clinic. The critical technology is spatial tracking and stereotactic orientation so that the operator can know the accurate imaging anatomical position of the surgical field, which has been proven to improve significantly the localization technique of neurosurgeons for surgical guidance and planning of the approach [3, 4]. However, with the progress of surgery, brain shift and brain deformation caused by the inevitable loss of cerebrospinal fluid and removal of lesions will lead to inaccurate localization and affect surgical accuracy [5]. Such image drift can only be corrected by real-time image scan compensation. Intraoperative CT and MRI can solve the above problems. However, they cannot be comprehensively popularized in clinical practice due to high equipment cost, long preoperative preparation time, long imaging time, radiation, significant space requirements, and other shortcomings [6, 7]. Fluorescence imaging is an innovative technique for visualizing tumor boundaries. 5-Aminolevulinic acid (or δ-aminolevulinic acid; 5-ALA), a natural heme precursor, is the commonly used fluorescence imaging agent for glioma. 5-AlA-induced fluorescence imaging can be used for tumor localization and boundary recognition and is standard care for high-grade gliomas(HGG) surgery [8]. However, the adverse reactions of 5-ALA may limit its application, including abnormal liver function, thrombocytopenia, anaphylaxis, respiratory disorders, etc [9]. At the same time, 5-ALA is not very robust and effective in delineating the boundaries of other non-HGG brain tumors [10]. Relevant studies have shown that only < 20% of low-grade gliomas(LGG) tumors showed visible fluorescence [11]. Fluorescein sodium is very effective in achieving maximum removal of HGG; however, it is only suitable for glioma and some highly aggressive metastases with ambiguous tissue boundaries and has poor imaging effects on other tumors [12]. Contrast-enhanced ultrasound(CEUS) can dynamically provide microvascular perfusion information of tissues in real-time, which improves the detection rate of lesions and differentiates benign and malignant lesions [13]. In recent years, several studies have focused on the CEUS characterization of different brain neoplasms and the effects of CEUS during the surgical procedure. Prada et al. [14] was the first study to use intraoperative CEUS to evaluate gliomas in 2014. By evaluating the CEUS images before and after resection of the lesions, they found that CEUS can help distinguish malignant and benign gliomas during surgery to modify operative strategies. Compared with B-mode ultrasound, CEUS can highlight the lesion and define the lesion boundary through vascular perfusion, which has a high specificity for identifying residual tumors to effectively improve the total resection rate [15–19].

Superb microvascular imaging (SMI) uses an adaptive algorithm to analyze the characteristics of motion artifacts, eliminate clutter signals, and realize low-speed microvascular visualization at a higher frame rate, higher spatial resolution, and with fewer motion artifacts without injecting contrast enhancement media [20]. SMI can operate in color SMI (cSMI) and monochrome SMI (mSMI). Smart 3D-SMI can reconstruct 3D images from 2D images to visualize 3D blood flow architecture. Multiple studies have shown that the diagnostic performance of SMI in diagnosing and evaluating thyroid, breast, kidney, liver, peripheral vascular, and other organ diseases is comparable to that of CEUS [21–25]. The SMI technique is also increasingly applied during neurosurgery before dural opening by using sterile sheets on the probe to explore and record B-mode morphology and vascular characteristics. Ishikawa et al. [26] took the lead in reporting 15 patients with brain tumors, depicted the SMI vascular characteristics of different pathological tumors, and found that SMI is beneficial for identifying tumor margins and distinguishing tumors from surrounding healthy tissues. Naritaka et al. [27] applied SMI to 11 cases of intracerebral hemorrhage surgery. By providing vascular information, SMI monitoring was helpful in identifying the extent of hematoma and residual lesions. However, the study of the application of SMI in brain tumors is relatively limited, and the value of SMI in tumor boundary demarcation and GTR needs to be further studied.

This study aimed to investigate whether SMI technology is helpful in achieving GTR through intraoperative application in the resection of different pathological brain tumors and to provide a theoretical basis for further research and application of SMI in neurosurgery.

## Materials and methods

### Patients and lesions

The Ethics Committee of Peking Union Medical College Hospital approved this prospective study. All patients understood and accepted the examination process and provided written informed consent. All patients underwent MRIs before the operation and within 48h after the operation. The inclusion criteria are as follows: (1) Complete imaging data, MRI examination within 48h before and after surgery, evaluation of the lesion by intraoperative grayscale ultrasound and SMI, and exclude cases where the open bone window is too small to obtain complete intraoperative ultrasound images or where it is challenging to display lesion boundaries; (2) The surgical purpose was to attempt to completely (100%) remove the enhanced tumor on MRI, while intracranial biopsy and palliative resection surgery were excluded; (3) The patient signs an informed consent form before surgery. From November 2020 to June 2022, 62 consecutive patients who underwent surgery in the neurosurgery department for brain tumors were included in this study. Three patients were excluded because they lacked complete ultrasound imaging data, and two patients were excluded because they underwent tumor biopsy only. Finally, a total of 57 patients were enrolled in the study.

### B-mode and SWI examinations

The navigation system recorded the tumor and important nerve function structure, made the surgical resection plan and approach, and determined the scalp and bone flap resection location with the help of the tip of the navigation stick probe. Ultrasonographic examinations were performed using an Aplio 500(L5-1, Canon, Tokyo, Japan). Before the operation, sterile saline was applied to the ultrasonic probe and placed on a sterile sleeve. After routine craniotomy according to the lesion site of the brain tumor and before opening the dura, the probe was gently placed on the surface of the dura for scanning by an experienced neurosurgeon.

Tumor size, depth, degree of edema, and tumor boundaries were evaluated on B-mode ultrasound, and tumor boundaries were evaluated under SMI conditions. The maximum diameter of the tumor was measured in the maximum section of the tumor, and the tumor size was divided into two categories: <5 cm and ≥ 5 cm. The nearest distance of the tumor perpendicular to the dura mater was defined as the depth of the tumor, and the tumor depth was divided into two categories: <2 cm and ≥ 2 cm. The width of peritumoral edema was measured in the most severe part of the edema, and the tumor depth was divided into two categories: <2 cm and ≥ 2 cm. Finally, the clarity of the tumor boundary was evaluated by conventional B-mode ultrasound and SMI mode, which were divided into clear and unclear. Each case was scanned in both mSMI and cSMI modes. Since mSMI is more sensitive to microflow than cSMI, using grayscale and mSMI dual-screen display to identify tumor boundaries, cSMI helps distinguish between microflows and calcification. An ultrasound doctor conducted the above assessments with five years of ultrasound experience. The clarity of the lesion’s boundary was divided into clear and unclear categories. Our analysis aimed to evaluate the clarity of the lesion boundary in B-mode and SMI and the correspondence between the two modes. An ultrasound doctor and a neurosurgeon with more than five years of clinical experience independently evaluated the clarity of the tumor boundary. If the classification of the above two doctors was inconsistent, it was decided by an ultrasound doctor with more than 15 years of clinical experience. The tumor boundary clarity data evaluated in B-mode and SMI mode of each patient were compared, and they were divided into the following three groups:

A: The use of SMI improved the definition of margins in lesions;
B: The quality of the tumor image was the same in the SMI and B mode;
C: The definition of the tumor was worse on SMI.

During the operation, after the dural incision, SMI was used to identify important vascular structures to avoid vascular damage. Throughout the entire surgical process, grayscale ultrasound and SMI could be used multiple times according to clinical needs for tumor localization and exploration of tumor boundaries, and further surgical resection was planned until the intraoperative evaluation was negative.

### Statistical analysis

Statistical software SPSS 22.0 (IBM Corp., Armonk, US). The Shapiro‒Wilk test was used to test the normality of the data, the nonparametric rank-sum test was used for statistical tests of hierarchical variables, the t-test was used for measurement data, and the Mann-Whitney U test was used for the evaluation of paired comparisons of enumeration data. Multivariate logistic regression analysis was used to screen the variables statistically significant to GTR. Cohen’s Kappa coefficient was used to analyze the consistency of the two doctors’ judgments on the boundary demarcation of brain tumors in the study subjects. For all statistical results, a P value < 0.05 indicated statistical significance.

## Results

The clinical and histological results of the 57 included patients are shown in Table 1. A total of 57 patients were enrolled in the study, including 32 males and 25 females, with an average age of 53.4 ± 14.1 years old (range 19 ~ 80). The 57 cases included 23 cases of high-grade glioma(HGG), 17 cases of low-grade glioma (LGG), 15 cases of metastasis, and 2 cases of meningioma. The maximum diameter of lesions was < 5 cm in 31 (54.4%) cases and ≥ 5 cm in 26 (45.6%) cases, and the depth of the lesion was > 2 cm in 44 (77.2%) cases and ≤ 2 cm in 13 (22.8%) cases. The frontal lobe was the most common anatomical area where lesions occurred (32, 56.1%). According to the assessment criteria of MRI before and within 48 h after the operation, 37 (63.9%) cases were classified as GTR, and 20 (35.1%) cases were classified as nGTR.

**Table 1:** Clinical characteristics of patients

Parameters	Value
Age	
Average	53.4 ± 14.1
Range	19 ~ 80
Sex, *N*(*N*,%)	
Female	25(43.9)
Male	32(56.1)
Tumors size (*N*,%)	
<5 cm	31(54.4)
≥5 cm	26(45.6)
Deep(cm) (*N*,%)	
<2 cm	44(77.2)
≥ 2 cm	13(22.8)
Tumor location (*N*,%)	
Frontal	32(56.1)
Temporal	9(15.8)
Parietal	4(7.0)
Frontoparietal	4(7.0)
Frontotemporal	3(5.3)
Occipital	2(3.5)
Parietooccipital	2(3.5)
Paracele	1(1.8)
Pathology (*N*,%)	
HGG	23(40.4)
LGG	17(29.8)
Metastasis	15(26.3)
Meningioma	2(3.5)
Extent of resection (*N*,%)	
Total	37(64.9)
Subtotal	20(35.1)
Total(*N*,%)	57(100.0)

### Consistency in the assessment of lesion boundary demarcation in B mode and SMI

The results showed that in the B-mode ultrasound, two doctors agreed on the demarcation of the lesion boundary in 48 patients and disagreed with the other nine patients. The inconsistent evaluations were defined as clear boundaries by the sonographer and blurred by the neurosurgeon. Cohen’s kappa coefficient of the two doctors was 0.860 (95% CI: 0.729–0.895, *P* < 0.001), with solid consistency. In the SMI, two doctors agreed on the demarcation of the lesion boundary in 53 patients, and the other four patients disagreed. Five cases were defined as clear boundaries by sonographers and blurred boundaries by neurosurgeons, and the other two cases were defined as the opposite. Cohen’s kappa coefficient of the two doctors was 0.684 (95% CI: 0.494–0.874, *P* < 0.001), with more substantial consistency.

### Comparison of tumor interface definition between B mode and SMI

**Fig. 1:**
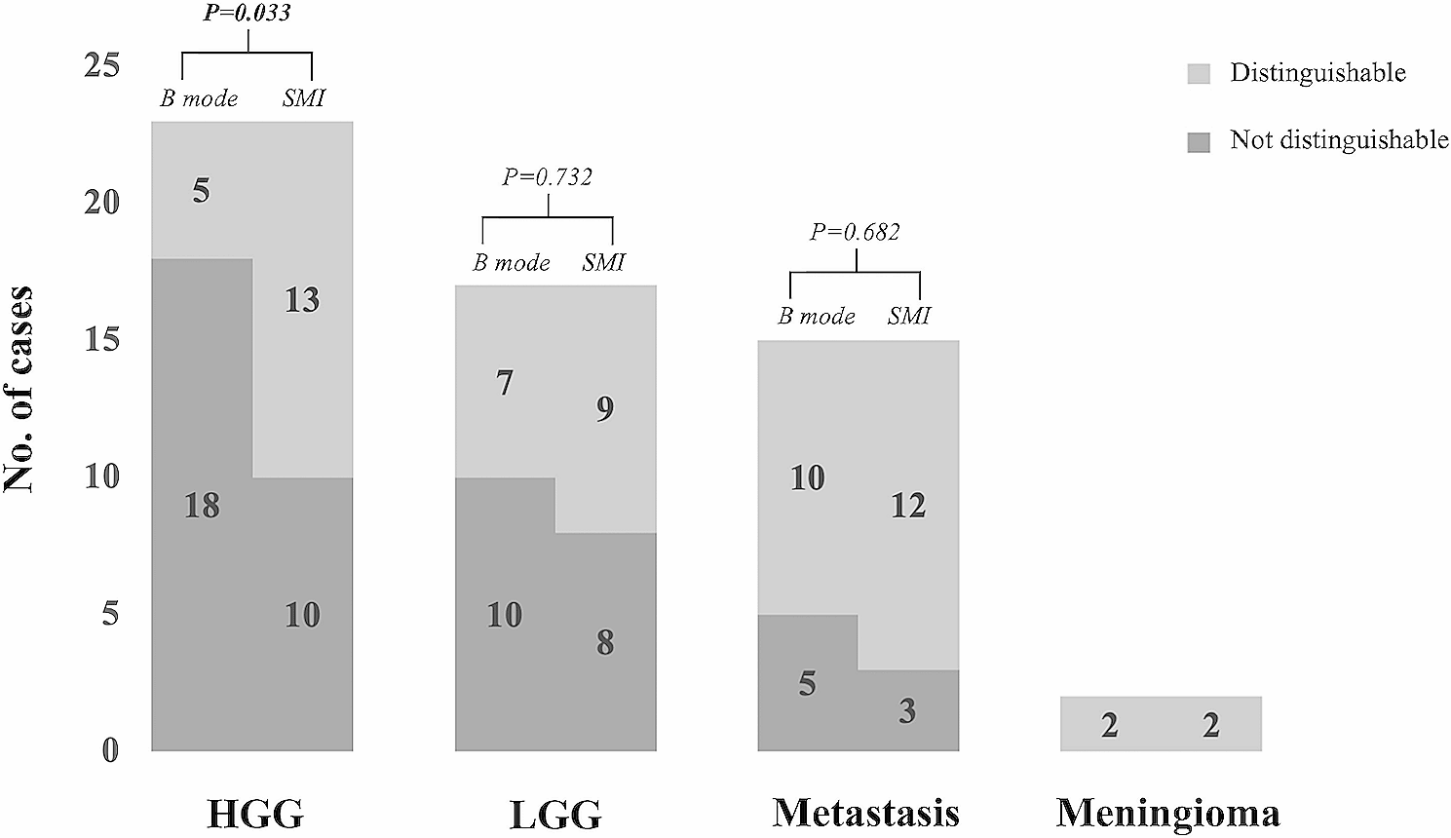
Comparison of tumor interface definition in B mode and SMI.

In 23 cases of HGG, B mode demonstrated 5 cases of well-defined boundaries, and SMI showed 13 cases of ill-defined boundaries, among which SMI improved 8 cases of lesion boundary recognition. There is a significant difference in the degree of the HGG interface definition between the B mode and SMI. In 17 cases of LGG, B mode demonstrated 7 cases of well-defined boundaries, and SMI showed 9 cases of ill-defined boundaries, among which SMI improved 2 cases of lesion boundary recognition. In 15 cases of metastasis, B mode demonstrated 10 cases of well-defined boundaries, and SMI showed 12 cases of ill-defined boundaries, among which SMI improved 2 cases of lesion boundary recognition. However, there was no significant difference in the degree of the LGG and metastasis interface definition between B mode and SMI. In both B mode and SMI, meningiomas showed well-defined boundaries(Fig. 1; Table 2).

**Table 2:** Compare the definition of margins between B-mode and SMI among all case

Pathology (*N*)	B mode vs. SMI in lesion definition		
	A	B	C
HGG (23)	8	15	0
LGG (17)	2	15	0
Metastasis (15)	2	13	0
Meningioma (2)	0	2	0

A: the use SMI improved the definition of margin in lesion; B: the quality of the tumor image was the same in the SMI and B mode; C: the definition of the tumor was worse in SMI

### Comparison of the clinical and ultrasonic characteristics between GTR and nGTR

In our study, 37 (63.9%) cases were classified as GTR, and 20 (35.1%) cases were classified as nGTR. Regarding clinical and ultrasound characteristics, there were no statistically significant differences in age, sex, case, tumor location, or peritumoral edema width. In terms of ultrasound characteristics, there were statistically significant differences in tumor size, depth, and delineation of tumor boundaries (*P* < 0.05)(Table 3). This study analyzed the influence of clinical and ultrasonic characteristics on GTR using binary logistic regression. Variables with *P* < 0.1 were included in the analysis, and the obtained Logistic model was statistically significant(χ2 = 26.228, *P* < 0.01). The model can correctly classify 80.7% of the subjects, with a sensitivity of 86.5% and a specificity of 70.0%. Age, tumor size, and lesion boundary clarity were statistically significant among the four independent variables in the model. The likelihood of GTR increased in patients with a tumor maximum diameter less than 5 cm compared with those with a tumor maximum diameter greater than 5 cm (OR = 12.317, 95%CI: 2.392–63.430). The likelihood of GTR increased when the tumor was most well-bounded versus poorly bounded (OR = 4.597, 95%CI:1.034–20.449).

**Table 3:** Clinical and ultrasound characteristics between GTR and nGTR.

Parameters(*N*)	Total resection	Subtotal resection	*P* value
Age			0.257
Average	55.0 ± 13.3	50.4 ± 15.3	
Range	19 ~ 80	25 ~ 79	
Sex(*N*,%)			0.492
Female	15(26.3)	10(17.5)	
Male	22(38.6)	10(17.5)	
Pathology(*N*,%)			0.536
HGG	15(26.3)	8(14.0)	
LGG	9(14.8)	8(14.0)	
Metastasis	11(19.3)	4(7.0)	
Meningioma	2(3.5)	0(0.0)	
Tumor location(*N*,%)			0.321
Frontal	23(40.4)	9(15.8)	
Temporal	5(8.8)	4(7.0)	
Parietal	2(3.5)	2(3.5)	
Frontoparietal	3(5.3)	1(1.8)	
Frontotemporal	1(1.8)	2(3.5)	
Occipital	2(3.5)	0(0.0)	
Parietooccipital	0(0.0)	2(3.5)	
Paracele	1(1.8)	0(0.0)	
Tumors size(*N*,%)			0.012*
<5 cm	24(42.1)	6(10.5)	
≥5 cm	13(22.8)	14(24.6)	
Deep(N,%)			0.023*
<2 cm	32(56.1)	12(21.1)	
≥ 2 cm	5(8.8)	8(14.0)	
Peritumor edema(*N*,%)			0.097
< 2 cm	25(43.9)	9(15.8)	
≥ 2 cm	12(21.1)	11(19.3)	
Tumor interface in SMI(*N*,%)			0.004*
Clear	21(49.1)	6(14.0)	
Unclear	8(15.8)	14(21.1)	
Total(*N*,%)	37(64.9)	20(35.1)	

*P<0.05

## Discussion

Common brain tumors include glioma, metastasis, meningioma, etc., and glioma is the most common intracranial malignant tumor. Cancer cells invade surrounding tissues by diverse modes of dissemination, such as expansive growth, collective invasion, mesenchymal migration, and amoeboid migration [28]. The most important hallmark of glioma is its aggressive behavior, and its extensive invasion of healthy brain tissue is the main reason for the poor prognosis and the difficulty in finding curative therapies [29]. In terms of the main invasion patterns of metastases, although the main pattern is well-demarcated growth(50%), vascular co-option(18%), and diffuse infiltration(32%) are also standard modes of tumor invasion. At the same time, there was no particular correlation between the different modes of invasion of brain metastases into the brain parenchyma and the primary tumor type [30]. Tumors arising in the dura, arachnoid, and pia meninges are called meningiomas, 80% benign. However, 20% of benign, atypical, and anaplastic meningiomas have pathophysiological manifestations of invasion to peripheral healthy tissues, and the postoperative recurrence rate is high [31–33].

Surgical resection is the primary treatment strategy, and EOR has been identified as a significant independent predictor of overall survival (OS) [34]. Specifically, for glioblastoma, GTR is directly related to OS. Lacroix et al. [35]found that the median survival time after primary surgery was improved by 4.2 months compared with the resection of 98% or more of the tumor volume being removed with or without resection. GTR has been identified as a target of brain tumor therapy, and every possible effort must be made to achieve maximum resection safely. The biological characteristics of brain tumors make it challenging for imaging to identify the exact lesion margin.

To achieve GTR, evaluation of tumor boundaries is essential. IOUS has been used for intraoperative navigation since 1980 [36]. IOUS is real-time, inherently convenient, distinctly cheaper, and has incomparable advantages over other imaging techniques [37]. This imaging method can contribute to accurately locating and delineating the boundary of the tumor, evaluating the extent of resection, and guiding the surgical approach, which has great significance for surgery [38]. The imaging information provided by IOUS gives neurosurgeons a new perspective to observe and study brain tumors. It will help to adjust the surgical strategy and, accurately guide tumor resection, maximize the EOR, and improve the safety of patients [39, 40]. Conventional two-dimensional ultrasound is affected by relatively low contrast and peritumoral edema of brain tumors, so it is challenging to define tumor boundaries.

**Fig. 2:**
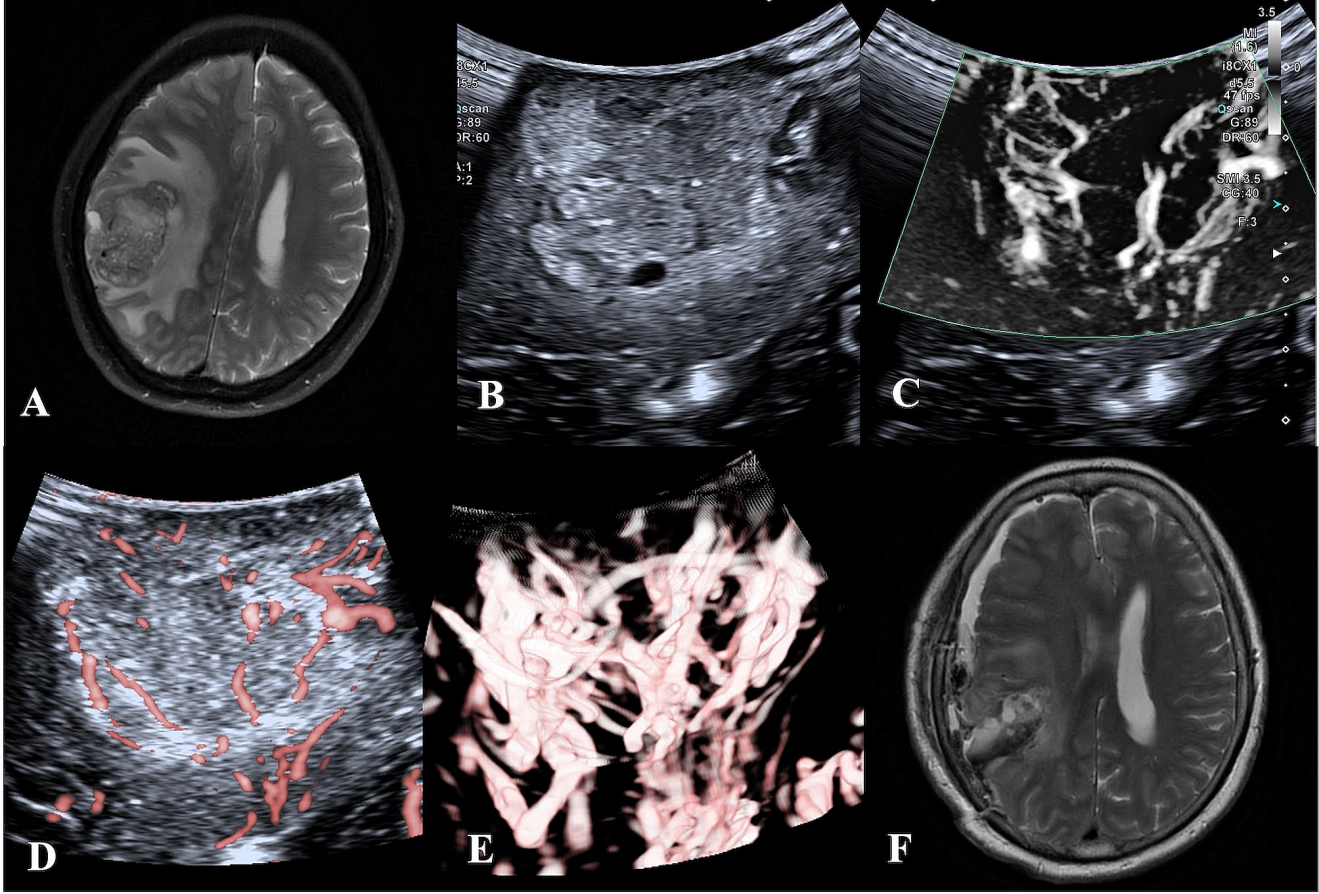
MRI, B-mode, and SMI of HGG in a 65-year-old woman. **(A)** Preoperative MRI T2W showed that the parietal lobe lesion showed high signal, irregular, blurred boundary, and large edema surrounding. **(B)** B-mode ultrasound, mixed echoes can be seen in the parietal lobe, irregular shape, unclear boundary, and peripheral edema. **B-D**. SMI mode, **B** is mSMI mode, **C** is cSMI mode, and **D** is Smart 3D-SMI mode. The vascular architecture in tumor parenchyma is extremely dilated, blood vessels are distorted and disordered, and the tumor boundary display is more transparent than that of B-mode ultrasound. **F**. Postoperative MRI T2W showed fluid and air accumulation in the operative area after resectioning parietal cystic lesions

**Fig. 3:**
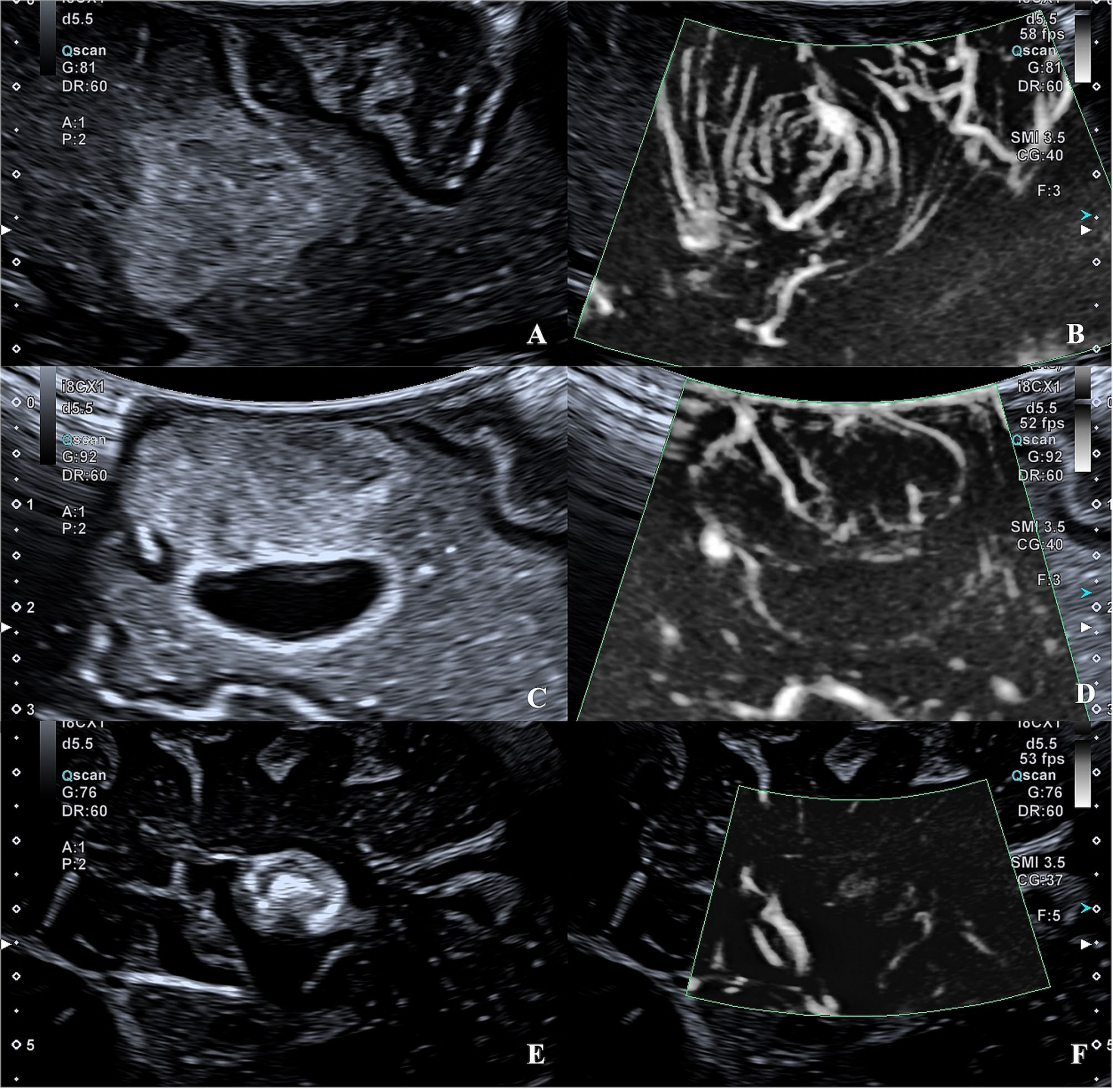
B-mode and SMI of LGG, metastasis and meningioma. The quality of the tumor image was the same as the definition of margins between B-mode and SMI. **A** and **B**, a 75-year-old male with low-grade glioma in the frontal lobe. **C** and **D**, a 65-year-old male with high-grade glioma in the frontal lobe. **E** and **F**, a 56-year-old female with meningioma in the paracele

CEUS enhances the display of microvessels through the injection of a contrast agent, which can dynamically display the blood flow of arteries, veins, and capillaries in real-time and reduce the signals from adjacent brain parenchyma [41]. CEUS can compensate for the disadvantages of conventional B-mode ultrasound, highlight the lesions and their margins, and contribute to distinguishing tumors from peritumoral edematous brain tissue [42]. SMI does not require the injection of contrast media but has shown comparable diagnostic efficacy in many other organ diseases. Our study shows that SMI can effectively compensate for B-mode ultrasound in identifying tumor margins for brain tumors, especially for HGG(Fig. 2). HGG is a highly vascularized tumor, and angiogenesis is one of the most apparent characteristics of HGG, which is in remarkable contrast to normal brain tissue [43]. In LGG and metastatic tumors, some tumor boundaries were more explicit under SMI, but there was no significant difference. In meningiomas, both B-mode ultrasound and SMI can identify the lesion boundary(Fig. 3). The peritumoral edema of LGG is significantly less than that of HGG, and the margin between the tumor and the adjacent normal brain is not covered by edema [44]. Metastases are highly heterogeneous, and the metastatic nature retains the inherent morphology of the primary tumor histologically. Usually, the lesion boundary is more precise than glioma in B-mode ultrasound morphology. Most are well-demarcated from the surrounding gliotic and rarefied brain parenchyma [45]. Meningiomas have been delineated in B-mode mode, but the application of SMI enables visualization of vascular modifications (such as destruction and remodeling) at the meningioma-brain interface.

In conclusion, it is a convenient, noninvasive, objective, and novel ultrasound Doppler technology. SMI does not reduce the quality of boundary imaging, especially for HGG boundary identification of highly aggressive tumors, which has a guiding significance for determining surgical plans and adjusting treatment strategies. At the same time, we also found greater consistency in assessing lesion boundaries under SMI conditions. Sonographers are better at recognizing and interpreting ultrasound images than neurosurgeons. When SMI strengthens the delineation of the lesion boundary, the lesion is more prominently displayed, and the consistency of interpretation by different doctors is improved to a certain extent.

In Table 4, we further analyzed the correlation between ultrasound features and GTR and found that the size and demarcation of the tumor were correlated with GTR. Munkvold et al. analyzed the correlation between intraoperative 3D B-mode ultrasound manifestations and GTR and found that in 144 gliomas, small tumors were significantly associated with GTR [46]. However, in our study, the demarcation between tumor and healthy brain tissue in SMI was significantly associated with GTR, mainly because SMI improves the delineation of brain tumors and provides histological information about vascularization, which can help neurosurgeons distinguish tumors from edema, further confirming the advantages of SMI over B-mode ultrasound in boundary delineation.

**Table 4:** Logistic regression analysis explored possible predictors of total resection

	OR(95%CI)	*P* value
Tumors size(cm)		0.003*
≥5 cm	1.00	
<5 cm	12.317(2.392–63.430)	
Deep(cm)		0.191
≥2 cm	1.00	
< 2 cm	3.275(0.553–19.389)	
Peritumor edema(cm)		0.147
≥ 2 cm	1.00	
< 2 cm	3.373(0.653–17.428)	
Tumor interface in SMI		0.045*
Unclear	1.00	
Clear	4.597(1.034–20.449)	

*P<0.05

As mentioned above, as an innovative Doppler technique in neurosurgery, SMI can make a sound judgment on the boundary of the tumor and observe the tumor in brain tumor resection. The internal and surrounding blood flow can guide the resection to a certain extent and help the patient achieve GTR as far as possible. With the continuous development of research and the continuous progress of ultrasound technology, intraoperative ultrasound, and new ultrasound technology will play an increasingly important role in neurosurgery.

This study also has some limitations. It is still necessary to expand the number of cases, extend the follow-up time, and compare the effects of different intraoperative auxiliary methods on patients’ quality of life and clinical outcome indicators. For the identification of residual tumors, intraoperative ultrasound detection of residual tumors and pathological biopsy are further research directions. Meanwhile, a new liquid acoustic coupler may become a new way to improve the identification of residual tumors by ultrasound.

## Data Availability

The data that support the findings of this study are available on request from the corresponding authors Jianchu Li and Hongyan Wang. The data are not publicly available because they contain information that could compromise research participant consent.

## References

[1] Tang S, Liao J, Long Y (2019). Comparative assessment of the efficacy of gross total versus subtotal total resection in patients with glioma: a meta-analysis. Int J Surg.

[2] Trevisi G, Barbone P, Treglia G, Mattoli MV, Mangiola A (2020). Reliability of intraoperative ultrasound in detecting tumor residual after brain diffuse glioma surgery: a systematic review and meta-analysis. Neurosurg Rev.

[3] Unsgaard G, Ommedal S, Muller T, Gronningsaeter A, Nagelhus Hernes TA (2002). Neuronavigation by intraoperative three-dimensional ultrasound: initial experience during brain tumor resection. Neurosurgery.

[4] Gerard IJ, Kersten-Oertel M, Hall JA, Sirhan D, Collins DL (2021). Brain shift in Neuronavigation of Brain tumors: an updated review of Intra-operative Ultrasound Applications. Front Oncol.

[5] Dorward NL, Alberti O, Velani B, Gerritsen FA, Harkness WF, Kitchen ND (1998). Postimaging brain distortion: magnitude, correlates, and impact on neuronavigation. J Neurosurg.

[6] Barbagallo GMV, Palmucci S, Visocchi M, Paratore S, Attina G, Sortino G (2016). Portable intraoperative computed tomography scan in image-guided surgery for Brain High-grade gliomas: analysis of Technical Feasibility and Impact on Extent of Tumor Resection. Oper Neurosurg (Hagerstown).

[7] Tsugu A, Ishizaka H, Mizokami Y, Osada T, Baba T, Yoshiyama M (2011). Impact of the combination of 5-aminolevulinic acid-induced fluorescence with intraoperative magnetic resonance imaging-guided surgery for glioma. World Neurosurg.

[8] Naik A, Smith EJ, Barreau A, Nyaeme M, Cramer SW, Najafali D (2022). Comparison of fluorescein sodium, 5-ALA, and intraoperative MRI for resection of high-grade gliomas: a systematic review and network meta-analysis. J Clin Neurosci.

[9] Schatlo B, Fandino J, Smoll NR, Wetzel O, Remonda L, Marbacher S (2015). Outcomes after combined intraoperative MRI and 5-aminolevulinic acid use in high-grade glioma surgery. Neuro Oncol.

[10] McCracken DJ, Schupper AJ, Lakomkin N, Malcolm J, Painton Bray D, Hadjipanayis CG (2022). Turning on the light for brain tumor surgery: a 5-aminolevulinic acid story. Neuro Oncol.

[11] Hosmann A, Millesi M, Wadiura LI, Kiesel B, Mercea PA, Mischkulnig M (2021). 5-ALA fluorescence is a powerful prognostic marker during surgery of low-Grade Gliomas (WHO Grade II)-Experience at two Specialized centers. Cancers (Basel).

[12] Sweeney JF, Rosoklija G, Sheldon BL, Bondoc M, Bandlamuri S, Adamo MA (2022). Comparison of sodium fluorescein and intraoperative ultrasonography in brain tumor resection. J Clin Neurosci.

[13] Ajmal S (2021). Contrast-enhanced Ultrasonography: review and applications. Cureus.

[14] Prada F, Mattei L, Del Bene M, Aiani L, Saini M, Casali C et al. Intraoperative cerebral glioma characterization with contrast-enhanced ultrasound. Biomed Res Int.2014;2014:484261. 10.1155/2014/484261 PMC4075093 25013784

[15] Prada F, Bene MD, Fornaro R, Vetrano IG, Martegani A, Aiani L (2016). Identification of residual tumor with intraoperative contrast-enhanced ultrasound during glioblastoma resection. Neurosurg Focus.

[16] Arlt F, Chalopin C, Muns A, Meixensberger J, Lindner D (2016). Intraoperative 3D contrast-enhanced ultrasound (CEUS): a prospective study of 50 patients with brain tumours. Acta Neurochir (Wien).

[17] Prada F, Del Bene M, Mauri G, Lamperti M, Vailati D, Richetta C (2018). Dynamic assessment of venous anatomy and function in neurosurgery with real-time intraoperative multimodal ultrasound: technical note. Neurosurg Focus.

[18] Arlt F, Chalopin C, Müns A, Meixensberger J, Lindner D (2016). Intraoperative 3D contrast-enhanced ultrasound (CEUS): a prospective study of 50 patients with brain tumours. Acta Neurochir.

[19] Della Pepa GM, Ius T, La Rocca G, Gaudino S, Isola M, Pignotti F (2020). 5-Aminolevulinic acid and contrast-enhanced Ultrasound: the combination of the two techniques to optimize the extent of resection in Glioblastoma surgery. Neurosurgery.

[20] Zhang Y, Sun X, Li J, Gao Q, Guo X, Liu JX (2022). The diagnostic value of contrast-enhanced ultrasound and superb microvascular imaging in differentiating benign from malignant solid breast lesions: a systematic review and meta-analysis. Clin Hemorheol Microcirc.

[21] Yang F, Wang C (2020). Consistency of superb microvascular imaging and contrast-enhanced ultrasonography in detection of intraplaque neovascularization: a meta-analysis. PLoS ONE.

[22] Park AY, Kwon M, Woo OH,Cho KR, Park EK, Cha SH, et al. A Prospective Study on the Value of Ultrasound Microflow Assessment to Distinguish Malignant from Benign Solid Breast Masses: Association between Ultrasound Parameters and Histologic Microvessel Densities. Korean J Radiol. 2019;20(5):759–772. 10.3348/kjr.2018.0515 PMC6470080 30993927

[23] Chen M, Fu X, Shen Y (2021). Evaluation of Multimode Color Doppler Flow Imaging in diagnosing solid renal tumor. Contrast Media Mol Imaging.

[24] Zhang L, Gu J, Zhao Y, Zhu M, Wei J, Zhang B (2020). The role of multimodal ultrasonic flow imaging in thyroid imaging reporting and Data System (TI-RADS) 4 nodules. Gland Surg.

[25] Lu R, Meng Y, Zhang Y, Zhao W, Wang X, Jin M (2017). Superb microvascular imaging (SMI) compared with conventional ultrasound for evaluating thyroid nodules. BMC Med Imaging.

[26] Ishikawa M, Ota Y, Nagai M, Kusaka G, Tanaka Y, Naritaka H (2017). Ultrasonography monitoring with superb microvascular imaging technique in brain tumor surgery. World Neurosurg.

[27] Naritaka H, Ishikawa M, Terao S, Kojima A, Kagami H, Inaba M (2020). Ultrasonographic superb microvascular imaging for emergency surgery of intracerebral hemorrhage. J Clin Neurosci.

[28] Friedl P, Alexander S (2011). Cancer invasion and the microenvironment: plasticity and reciprocity. Cell.

[29] Seifert, Swanson KR, Hawkins-Daarud, Klink H (2017). The biology and mathematical modelling of glioma invasion: a review. J R Soc Interface.

[30] Berghoff AS, Rajky O, Winkler F, Bartsch R, Furtner J, Hainfellner JA (2013). Invasion patterns in brain metastases of solid cancers. Neuro Oncol.

[31] Al-Rashed M, Foshay K, Abedalthagafi M (2019). Recent advances in Meningioma Immunogenetics. Front Oncol.

[32] Lee YS, Lee YS (2020). Molecular characteristics of meningiomas. J Pathol Transl Med.

[33] Shao Z, Liu L, Zheng Y, Tu S, Pan Y, Yan S et al. Molecular mechanism and Approach in Progression of Meningioma. Front Oncol.2020;10:538845. 10.3389/fonc.2020.538845 PMC7518150 33042832

[34] Giammalva GR, Ferini G, Musso S, Salvaggio G, Pino MA, Gerardi RM (2022). Intraoperative Ultrasound: Emerging Technology and Novel applications in brain tumor surgery. Front Oncol.

[35] Lacroix M, Abi-Said D, Fourney DR, Gokaslan ZL, Shi W, DeMonte F (2001). A multivariate analysis of 416 patients with glioblastoma multiforme: prognosis, extent of resection, and survival. J Neurosurg.

[36] Rubin JM, Mirfakhraee M, Duda EE, Dohrmann GJ, Brown F (1980). Intraoperative ultrasound examination of the brain. Radiology.

[37] Sabeghi P, Zarand P, Zargham S, Golestany B, Shariat A, Chang M (2024). Advances in Neuro-Oncological imaging: an update on Diagnostic Approach to Brain tumors. Cancers (Basel).

[38] Pichardo-Rojas PS, Zarate C, Arguelles-Hernández J, Barrón-Lomelí A, Sanchez-Velez R, Hjeala-Varas A (2024). Intraoperative ultrasound for surgical resection of high-grade glioma and glioblastoma: a meta-analysis of 732 patients. Neurosurg Rev.

[39] Xiong Z, Luo C, Wang P, Hameed NUF, Song S, Zhang X, Wu S, Wu J, Mao Y (2022). The intraoperative utilization of Multimodalities could improve the prognosis of adult glioblastoma: a single-Center Observational Study. World Neurosurg.

[40] Pino MA, Imperato A, Musca I, Maugeri R, Giammalva GA-O, Costantino G (2018). New Hope in Brain Glioma surgery: the role of intraoperative Ultrasound. A review. Brain Sci.

[41] Sidhu PS, Cantisani V, Dietrich CF, Gilja OH, Saftoiu A, Bartels E (2018). The EFSUMB guidelines and recommendations for the clinical practice of contrast-enhanced ultrasound (CEUS) in non-hepatic applications: update 2017 (extended version). Ultraschall Med.

[42] Prada F, Perin A, Martegani A, Aiani L, Solbiati L, Lamperti M (2014). Intraoperative contrast-enhanced ultrasound for brain tumor surgery. Neurosurgery.

[43] Ahir BK, Engelhard HH, Lakka SS (2020). Tumor Development and Angiogenesis in Adult Brain Tumor: Glioblastoma. Mol Neurobiol.

[44] Petridis AK, Anokhin M, Vavruska J, Mahvash M, Scholz M (2015). The value of intraoperative sonography in low-grade glioma surgery. Clin Neurol Neurosurg.

[45] Takei H, Rouah E, Ishida Y (2016). Brain metastasis: clinical characteristics, pathological findings and molecular subtyping for therapeutic implications. Brain Tumor Pathol.

[46] Munkvold BKR, Jakola AS, Reinertsen I, Sagberg LM, Unsgård G, Solheim O (2018). The Diagnostic properties of Intraoperative Ultrasound in Glioma Surgery and Factors Associated with Gross Total Tumor Resection. World Neurosurg.

